# Legal protection of traditional medicine knowledge in Indonesia: integrating local wisdom and modern regulations

**DOI:** 10.11604/pamj.2025.51.73.47755

**Published:** 2025-07-11

**Authors:** Umaira Hayuning Anggayasti, Marzellina Hardiyanti, Aga Natalis

**Affiliations:** 1 Faculty of Law, Universitas Diponegoro, Jalan dr. Antonius Suroyo, Tembalang, Semarang City, Central Java, Indonesia

**Keywords:** Traditional knowledge, intellectual property law, pancasila, legal pluralism, biodiversity conservation

## To the editors of the Pan African Medical Journal

Characterized by its agrarian nature, Indonesia possesses a variety of natural resources frequently utilized as sources for traditional medicines. Indonesia's extensive forest resources, spanning 34,624,957 hectares, present significant potential and advantages derived from forest products. Indonesia is the second most biodiverse country globally, followed by Brazil, featuring around 30,000 plant species. Approximately 9,600 are recognized for their potential medicinal properties [1]. The daily lives of Indonesians reflect a significant reliance on plant-based traditional medicines, which serve not merely as alternatives to modern medicine but also as essential components of cultural heritage that continue to hold substantial belief in contemporary society [2].

The notion of traditional medicinal knowledge in Indonesia has the potential to contribute significantly to national prosperity [3]. Acknowledging and improving the values inherent in traditional medicinal knowledge is likely to fortify identity and increase the application of traditional medicine, thereby contributing to the attainment of social and developmental objectives [4].

International dialogue has led to significant agreements concerning the safeguarding of traditional medicinal knowledge, specifically the Convention on Biological Diversity (CBD) and the Nagoya Protocol on Access to Genetic Resources and the Fair and Equitable Sharing of Benefits Arising from Their Utilisation (Nagoya Protocol) [5]. In light of these two agreements, it is evident that the forum lacks definitive regulations concerning traditional medicinal knowledge.

In Indonesia, the inadequacy of the Intellectual Property Rights Law in offering protection for traditional medicine knowledge is evident [6]. The protection of intellectual property for traditional knowledge is considered cumbersome and not entirely suitable, as the Patent Law specifically excludes methods related to examination, treatment, cure, and/or surgery from patent applications, which effectively bars the patenting of traditional healing practices. Furthermore, there exists a degree of ambiguity and overlap in the regulations across multiple laws, including the Plant Variety Protection Law, the Copyright Law, the Patent Law, the Cultural Advancement Law, and Government Regulation No. 56 of 2022 concerning Community Intellectual Property. The regulations primarily emphasize economic factors and do not adequately address the comprehensive protection of traditional knowledge, particularly about spiritual values and cultural identity [7].

Article 28I Paragraph (3) of the 1945 Constitution of the Republic of Indonesia asserts the importance of respecting cultural identity and the rights of traditional communities, aligning this respect with the progression of contemporary society and civilization. Therefore, traditional knowledge, which forms the basis of cultural identity and the rights of indigenous communities, is implicitly acknowledged as a constitutional right within the Indonesian state and is integrated into the framework of human rights [8].

The use of traditional medicinal plants in Indonesia is deeply embedded in cultural diversity and local knowledge, which combines communal beliefs with scientific evidence facilitated by advancements in modern technology. Empirical studies increasingly validate specific conventional healing methods. Government Regulation No. 56 of 2022 on Community Intellectual Property, specifically Article 8 (i), acknowledges that knowledge related to healing, traditional medicine, and healing practices is classified as traditional knowledge deserving of legal protection.

The legal safeguarding of traditional knowledge, particularly in traditional medicine, represents a synthesis of indigenous wisdom and national legislation, which can be associated with the theory of legal pluralism. Menski [9] asserts that legal pluralism within the Indonesian context emerges as a historical and sociological phenomenon shaped by the rich diversity of cultures, ethnic groups, languages, and religions in society. Consequently, the political essence of law concerning the conservation of biological resources should acknowledge the local knowledge of customary law communities as a normative framework and as a source of legal material in conservation legislation, ensuring that these communities remain connected to their natural environment [10].

Alongside Indonesia, India possesses a robust heritage of using traditional medicinal plants. About 65% of India's population relies solely on local knowledge systems regarding medicinal plants, whereas 70% reside in villages where access to and affordability of modern allopathic medicine are restricted. Traditional, Complementary, and Alternative Medicine (TCAM) is significant, encompassing classical systems like Ayurveda, Unani, and Siddha, which trained practitioners, established texts, and formal state acknowledgment. Ayurveda and Siddha emerged over 3,000 years ago in northern and southern India, whereas Unani, which has its roots in Greece, became more widely recognized after 1969. Despite the limited official support, folk medicinal knowledge has been maintained through oral transmission within villages [11].

In South Tyrol, the application of traditional medicinal plants is characterized primarily by the prevalence of herbal species, with woody species following in significance. In contrast, the utilization of ferns, mushrooms, and lichens remains comparatively restricted. The primary element in this region is cultivated medicinal plants, which mitigates the risk of overexploitation typically observed in natural mountainous areas like the Himalayas or the Ethiopian Highlands, making it a non-issue in South Tyrol. The findings align with reports from other areas with a longstanding history of utilizing medicinal plants, including Central China and the Balkan Mountains. Plant species classified as vulnerable but remain unprotected necessitate additional focus, either by being incorporated into South Tyrolean legislation or by executing ecosystem restoration initiatives [12].

The presence of traditional medicinal plant products from the two aforementioned countries drives Indonesia to enhance the safeguarding of intellectual property rights related to traditional medicinal plants. This involves integrating local wisdom within traditional medicine, which is a manifestation of traditional culture and represents a heritage that requires preservation and protection for its continued sustainability.
